# From stability to dynamics: understanding molecular mechanisms of regulatory T cells through *Foxp3* transcriptional dynamics

**DOI:** 10.1111/cei.13194

**Published:** 2018-09-17

**Authors:** D. Bending, M. Ono

**Affiliations:** ^1^ Institute of Immunology and Immunotherapy, College of Medical and Dental Sciences University of Birmingham UK; ^2^ Department of Life Sciences, Faculty of Natural Sciences Imperial College London London UK

**Keywords:** Foxp3, Nr4a3, regulatory T cells (T_reg_), Time of cell kinetics and activity (Tocky), transcriptional autoregulatory circuit

## Abstract

Studies on regulatory T cells (T_reg_) have focused on thymic T_reg_ as a stable lineage of immunosuppressive T cells, the differentiation of which is controlled by the transcription factor forkhead box protein 3 (Foxp3). This lineage perspective, however, may constrain hypotheses regarding the role of Foxp3 and T_reg_ ***in vivo***, particularly in clinical settings and immunotherapy development. In this review, we synthesize a new perspective on the role of ***Foxp3*** as a dynamically expressed gene, and thereby revisit the molecular mechanisms for the transcriptional regulation of ***Foxp3***. In particular, we introduce a recent advancement in the study of Foxp3‐mediated T cell regulation through the development of the Timer of cell kinetics and activity (Tocky) system, and show that the investigation of ***Foxp3*** transcriptional dynamics can reveal temporal changes in the differentiation and function of T_reg_ ***in vivo***. We highlight the role of ***Foxp3*** as a gene downstream of T cell receptor (TCR) signalling and show that temporally persistent TCR signals initiate ***Foxp3*** transcription in self‐reactive thymocytes. In addition, we feature the autoregulatory transcriptional circuit for the ***Foxp3*** gene as a mechanism for consolidating T_reg _differentiation and activating their suppressive functions. Furthermore, we explore the potential mechanisms behind the dynamic regulation of epigenetic modifications and chromatin architecture for ***Foxp3*** transcription. Lastly, we discuss the clinical relevance of temporal changes in the differentiation and activation of T_reg_.

## Introduction

### Dynamics of Foxp3 transcription as a key to understanding regulatory T cell‐mediated immune regulation

It is widely considered that regulatory T cells (T_reg_) constitute a distinct lineage of CD4^+^ T cells dedicated for immunosuppression 1. Key evidence for the distinct lineage include: (i) T_reg_ development is controlled by the transcription factor *Foxp3* 2; and (ii) the development of T_reg_ in the thymus is delayed to after that of other T cells under physiological conditions 3. However, accumulating evidence shows the simultaneous development of T_reg_ and other T cells 4, 5 and T_reg_ plasticity is now widely recognized, as T_reg_ can lose forkhead box protein 3 (Foxp3) expression and become effector T cells (ex‐T_reg_) during inflammation 6, 7. Thus, studies on dynamic changes in the differentiation and activation status of T_regs_ – and other T cells – *in vivo* is essential for understanding Foxp3‐mediated T cell regulation. This dynamic perspective is important not only for basic research but also clinical research and immunotherapy development, which is illustrated by the catastrophic clinical trial of the superagonistic anti‐CD28 antibody TGN1412 in 2006.

TGN1412 was developed as an immunosuppressive treatment after an anti‐CD28 antibody was found to suppress autoimmune reactions in rodent models 8. TGN1412 was thus designed to bind to the CD28 molecule on the surface of T_reg_ which would, in turn, theoretically suppress non‐T_regs_ 9. This trial, however, resulted in catastrophe, where all six volunteers given TGN1412 developed a ‘cytokine storm’ due to stimulation of a significant proportion of T cells 10. Later, it was found that CD28 molecules in memory‐phenotype T cells are down‐regulated in primates – which does not occur in humans – and this species difference was deemed to be the major cause of the incident 11. Meanwhile, Vitetta and Ghetie pointed out that T_regs_ and non‐T_regs_ may not represent strictly separate lineages, and therefore the assumption of specific activation of T_regs_ may have been inappropriate 12. In fact, basic studies later showed the plasticity of T_regs_: T_regs_ may lose Foxp3 expression during inflammation and non‐T_regs_ may acquire Foxp3 expression 13. Summarizing, the case provides two important lessons: first, the concepts of lineage stability may constrain hypotheses, which can be detrimental in clinical settings; and secondly, it is fundamental to investigate the dynamic changes in the differentiation and activation statuses of T_regs_ and other T cells *in vivo*, which are still poorly understood.

The key evidence of Foxp3 as the lineage‐specification transcription factor is that mutations in the *Foxp3/FOXP3* gene can lead to autoimmune disease in both mice 14 and humans 15. However, this does not preclude the dynamic induction of Foxp3 as a negative regulator in response to T cell activation. In fact, Foxp3 expression can be induced solely by T cell receptor (TCR) signals in human T cells 16 and, although less efficiently, also in mice 17, and the induction is enhanced by transforming growth factor (TGF)‐β and interleukin (IL)‐2 18. TGF‐β is produced by activated antigen‐presenting cells such as dendritic cells 19 and macrophages 20, while IL‐2 is produced mainly by activated T cells, particularly CD4^+^ T cells 21. As the immunosuppressive T_reg_ population is commonly identified by the expression of Foxp3 (as Foxp3^+^ T cells in mice 2 and Foxp3^high^CD45RA^+^ 22, 23 or Foxp3^+^CD127^–^CD25^high^ T cells 24, 25 in humans), the investigation of *Foxp3* dynamics *in vivo*, especially during immune responses, will be key for understanding the *in‐vivo* dynamics of T_reg_ and T cell regulation. To this end, we have recently developed a new technology, the Timer of cell kinetics and activity (Tocky) system, which allows the investigation of *invivo *dynamics of Foxp3 and T_regs_ during physiological immune responses 26, 27.

In this paper, we will aim to introduce a dynamic perspective to the molecular mechanisms that account for the transcriptional and epigenetic control of the *Foxp3* gene, and thereby to improve the understanding of Foxp3‐mediated T cell regulation *in vivo*.

### Development of Tocky for investigating *in‐vivo* dynamics of T_reg_ differentiation

The current understanding of T_reg_ differentiation and function is based significantly on evidence obtained by *Foxp3* fluorescent protein (FP) reporters such as enhanced green fluorescent protein (EGFP) 28, 29 and fate mapping systems for the *Foxp3* gene (e.g. *Foxp3^CreGFP^*:*Rosa26^RFP^* 17 and *Foxp3^ERT2CreGFP^*:*Rosa26^YFP^* 30). Notably, all these systems rely upon stable FPs such as GFP, the half‐life of which is longer than 56 h. Therefore, temporal changes in *Foxp3* transcription shorter than 2–3 days cannot be investigated by these reporter systems.

**Figure 1:**
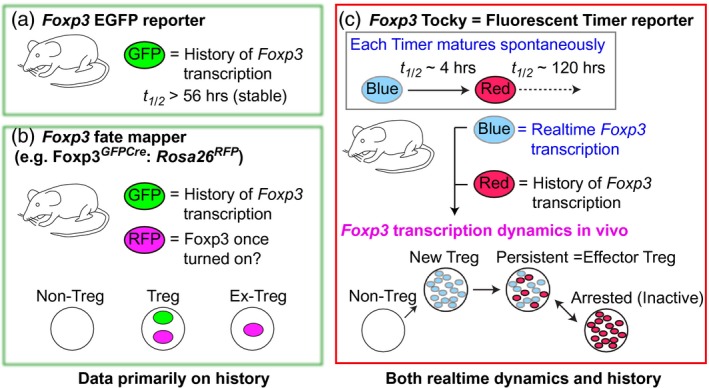
Comparison of tools to investigate forkhead box protein 3 (Foxp3)‐expressing T cells *in vivo*. (a) Most *Foxp3* reporter mice use stable fluorescent proteins (FP), such as enhanced green fluorescent protein (EGFP), the half‐life of which is > 56 h. (b) Foxp3 fate mappers such as *Foxp3^GFPCre^*:*Rosa26^RFP^* allow the identification of regulatory T cells (T_regs_) with Foxp3 expression and ex‐T_regs_ that lost Foxp3 expression. Notably, both GFP and red fluorescent protein (RFP) are stable FPs. (c) *Foxp3*–Tocky uses fluorescent timer, the emission spectrum of which changes spontaneously and irreversibly from blue to red fluorescence. The half‐life of blue fluorescence is ~4 h, and thus reports the ‘real‐time’ activity of *Foxp3* transcription. In contrast, the half‐life of red fluorescence is ~120 h and thus reports the history of *Foxp3* transcription. The Tocky system combines blue and red fluorescence data and identifies characteristic transcriptional dynamics including new, persistent and arrested (inactive).

In order to understand the *in‐vivo* dynamics of those molecular mechanisms underlying the differentiation and function of T_regs_, we have recently developed the Tocky system using Fluorescent Timer protein (Timer). Timer proteins exhibit a short‐lived blue fluorescent form, before maturation to the stable red state 27, 31. The half‐life of blue fluorescence is ~ 4 h 26, 27 and that of the mature red fluorescence is ~ 5 days 26. Thus, blue and red fluorescence (blue and red) provide a measurement of both the ‘real‐time’ activity and the history of gene transcription 26. Tocky uses this information to analyse dynamic changes quantitatively in transcriptional activities during cellular activation and differentiation 27. Importantly, we have identified three characteristic dynamics of transcription in the Tocky system: blue^+^red^– ^cells are those that have just initiated transcription (new); blue^+^red^+^ cells along the diagonal line between blue and red axes are those with sustained transcription, accumulating both blue and red form proteins (persistent); and blue^–^red^+^ cells are those that have recently down‐regulated gene expression under the detection threshold of flow cytometry and are inactive in transcription of the gene (arrested or inactive) 27 (Fig. 1).

**Figure 2:**
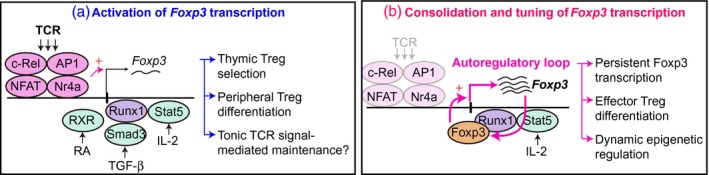
Activation *versus* consolidation and tuning of *Foxp3* transcription. We propose to classify *Foxp3* transcriptional regulation into two major mechanisms. (a) Activation of *Foxp3* transcription is regulated mainly by T cell receptor (TCR) signals and enhanced by interleukin (IL)‐2, transforming growth factor (TGF)‐β and retinoic acid (RA). This may lead to thymic regulatory T cell (T_reg_) selection and peripheral T_reg_ differentiation. In addition, tonic TCR signals through self‐reactive TCRs may use this mechanism to regulate homeostatic *Foxp3* transcription. (b) Consolidation and tuning of *Foxp3* transcription. The maintenance of *Foxp3* transcription requires CNS2 of the *Foxp3* gene, which may provide a platform for Foxp3–Runx1/CBF‐β complex to form the autoregulatory transcriptional circuit (autoregulatory loop) for the *Foxp3* gene. The activity of this loop can be affected by IL‐2 signalling via phosphorylated signal tranducer and activator of transcription‐5 (STAT)‐5. This mechanism may lead to temporally persistent *Foxp3* transcription, which promotes effector T_reg_ differentiation, and the dynamic regulation of epigenetic modifications during T_reg_ differentiation.

*Foxp3* transcription is controlled mainly by 5' upstream sequences and conserved non‐coding sequences (CNS) 1–3 in intronic regions 7, 32, 33, 34. Importantly, while TCR signals (together with TGF‐β and IL‐2 signals) induce Foxp3 expression in any T cells *in vitro* 18, naturally arising Foxp3 expression is found mainly in self‐reactive T cells in non‐inflammatory conditions 1. Thus, we will classify the mechanisms for *Foxp3* transcription into two groups, as follows:

1. Mechanisms for the activation of *Foxp3* transcription: these are used during thymic T_reg_ selection and peripheral T_reg_ differentiation and are potentially involved in the mechanism for tonic TCR signal‐mediated activation of *Foxp3* transcription.
2. Mechanisms for the consolidation and tuning of *Foxp3* transcription: these are used for sustaining *Foxp3* transcription over time, which induces effector T_reg_ differentiation and the dynamic regulation of epigenetic modifications, such as demethylation of CpG islands in enhancer regions (Fig. 2).

### Mechanisms for the activation of *Foxp3* transcription

#### Foxp3 as a TCR signal downstream gene

The differentiation and function of T_regs_ is under the control of TCR signals 35, 36, 37, 38. In the thymus, the recognition of cognate antigen induces not only negative selection but also the differentiation of CD25^+^Foxp3^+^ T_regs_ from CD4‐SP cells using transgenic TCR systems 39, 40, 41. Conversely, TCR transgenic mice in the recombination activating gene (*Rag*)‐deficient backgrounds lack Foxp3^+^ T cells due to the absence of self‐antigen recognition 42, 43. The analysis of TCR signals using reporter mice have provided insights into the mechanism for TCR‐mediated T_reg_ differentiation. The Hogquist group showed that T_regs_ receive strong TCR signals in the thymus and the periphery when analysed using a Nur77(Nr4a1)‐GFP transgenic reporter 44. Using *Nr4a3*–Tocky, we have shown that Foxp3 expression in the thymus occurs in T cells that have received temporally persistent TCR signals 27. Furthermore, using *Foxp3*–Tocky we showed that *Foxp3* transcription is initiated in non‐T_reg_ cells during inflammation in the periphery 26. In humans, activation‐induced Foxp3 in conventional T cells suppresses their proliferation and cytokine production in a cell‐intrinsic manner 45. In addition, activated conventional T cells can express both Foxp3 and cytotoxic T lymphocyte antigen‐4 (CTLA‐4), and thereby acquire the suppressive function that is dependent upon CTLA‐4 46. These suggest that Foxp3 has a role in negative feedback regulation of T cell activation in co‐operation with other immunoregulatory molecules, including CTLA‐4. *Foxp3* transcription, therefore, is thus under the control of TCR signals in both the thymus and the periphery. In addition, in normal homeostasis, T_regs_ and naturally arising memory‐phenotype T cells are self‐reactive and receive ‘tonic’ TCR signals in the periphery 27, 44. Considering this evidence, the biological meaning of TCR signal‐induced Foxp3 expression includes two situations: (i) antigen recognition‐induced *Foxp3* transcription in Foxp3^–^ cells (conventional T cells; non‐T_regs_) in the thymus and the periphery; and (ii) the effects of tonic TCR signals in Foxp3^+^ T_regs_.

In line with the evidence of Foxp3 expression upon TCR stimulation, the gene regulatory regions of the *Foxp3* gene are bound by transcription factors downstream of major branches of the TCR signalling pathway, including nuclear factor of activated T cells (NFAT) and activator protein 1 (AP1) 47, the nuclear factor kappa B (NF‐B) components c‐Rel and p65 32, 48, 49, 50, cyclic AMP response element‐binding protein (CREB) 51 and Nr4a proteins 52 (Fig. 2).

Nr4a proteins (Nr4a1, Nr4a2 and Nr4a3) bind to their target sequences as homodimers or heterodimers and regulate transcription 53, 54. Foxp3^+^ T_reg_ differentiation is abolished in Nr4a1/2/3 triple knock‐out (KO) mice and Nr4a1/3 double KO, and these mice develop fatal autoinflammatory disease 52. Nr4a proteins bind to the *Foxp3 *promoter upon anti‐CD3 stimulation 52 and retroviral gene transduction of *Nr4a2* or *Nr4a3* induces *Foxp3* transcription 55. Importantly, however, Nr4a triple KO lack not only Foxp3^+^ T_regs_ but also most of the double‐positive (DP) cell population 52, which suggests that the T_reg_ reduction in these KO mice is a consequence of defective regulation of positive and negative selection. Meanwhile, we have identified *Nr4a3* as the gene that is the most correlated with the effects of TCR signals in the thymus and the periphery, followed by *Nr4a1* 27. Specifically, using canonical correspondence analysis (CCA) 56, we analysed the transcriptome data set of thymic T cell populations and that of resting and anti‐CD3 stimulated peripheral T cells, and thereby identified the genes that were correlated with both thymic T cells under selection (*in‐vivo* TCR signals) and peripheral T cell activation 27. By developing *Nr4a3*–Tocky, we have shown that temporally persistent TCR signals sustain *Nr4a3* transcription and initiate *Foxp3* transcription 27. This leads to the new model for Nr4a, that the recognition of cognate antigen conveys persistent TCR signals, which induce and accumulate Nr4a proteins and thereby control thymic selection and differentiation processes including T_reg_ differentiation.

#### *Foxp3 *transcription‐enhancing cytokine signals

*Foxp3* transcription is activated by IL‐2 signalling in the presence of TCR stimulation and TGF‐β signalling 18. However, it is unknown whether these cytokine signals can regulate *Foxp3* transcription independently from TCR signalling.

IL‐2 signalling is a central cytokine for T cell activation, proliferation and differentiation 21. The expression of CD25 (IL‐2R α‐chain) is induced by TCR and CD28 signals and forms the high‐affinity IL‐2R, together with IL‐2R β ‐chain (CD122) and the common ‐chain (CD132) 57, 58. IL‐2 binding to IL‐2R triggers phosphorylation of signal transducer and activator of transcription (STAT)‐4 and STAT‐5 by the associated kinases Janus kinase (Jak)1 and Jak3, which promote cell cycle entry and proliferation of TCR‐stimulated T cells 59. In addition to the role in T cell activation, CD25 is also a surface marker for T_reg_ in mice 60 and humans 61. In fact, IL‐2 signalling is functional in T_regs_. Phosphorylated STAT‐5 binds to the promoter and CNS2 and activates *Foxp3* transcription 62, 63. KO mice for the genes that are involved in IL‐2 signalling (*Il2* 64, *Il2ra* 64, *Il2rb* 65, *Jak3* 66 and *Stat5a*/*Stat5b* 67) have reduced Foxp3^+^ T cells in the thymus and periphery. Thus, IL‐2 signalling is required for the activation of *Foxp3* transcription, most probably during both the early phase of T_reg_ differentiation as well as the maintenance of both *Foxp3* transcription and the T_reg_ population. Considering the primary role of IL‐2 for the activation and proliferation of T cells 21, this suggests a role of *Foxp3* as a sensor for the IL‐2 abundance in the environment surrounding individual T cells. In other words, when T cells are activated IL‐2 becomes abundant, which enhances Foxp3 expression in nearby T cells. Given that IL‐2R expression in T_regs_ absorbs IL‐2 and suppresses IL‐2‐mediated T cell proliferation 68, the size of the T cell population may be self‐regulated through the feedback mechanism involving IL‐2, CD25 and Foxp3 38.

TGF‐β signalling has multi‐faceted effects on tissue development and regeneration, inflammation and cancer in a context‐dependent manner 69. The importance of TGF‐β signalling in T cells is recognized particularly in mucosal and tumour immunity 70. The transcriptional response of T cells to TGF‐β signalling is also context‐dependent, and is illustrated by the reciprocal differentiation of T helper type 17 (Th17) and T_regs_ by IL‐6 and IL‐2, respectively, in the presence of TGF‐β 71, 72. TGF‐β signal‐activated Mothers Against DPP Homologue 3 (SMAD3) binds to the CNS1 of the *Foxp3* gene 32, 73. However, the genetic deletion of the SMAD‐binding site does not change the frequencies of T_regs_ in the thymus and the periphery, apart from marginal reductions of Foxp3^+^ T cells in Peyer’s patches and lamina propria in aged mice 74. This suggests that TGF‐β controls *Foxp3* transcription through multiple sites in the *Foxp3* gene and/or through the induction of other factors. While IL‐2 signalling is intrinsically required for T_reg_ differentiation, as discussed above, the opposing effects of IL‐6 signalling seem to be reactive and inflammation‐dependent, as the genetic deletion of *Stat3* does not affect T_reg_ populations, while inhibiting the differentiation of T_regs_ in the CD45RB^high^ T cell‐mediated colitis model 75.

Veldhoen and Stockinger have proposed the model that TGF‐β skews CD4^+^ T cell differentiation from Th1 to Th17 76, and as such, TGF‐β may shift T cells from the Th1–Th2 axis to the Th17–T_reg_ axis. In the TGF‐β‐rich microenvironment, such as in the intestines, tumour or damaged tissues undergoing regeneration and remodelling, the persistence of pathogen or autoantigen may activate monocytes and dendritic cells, and thereby repress *Foxp3* transcription and promote Th17 differentiation, as observed in rheumatoid arthritis patients 77. In contrast, once the activation of innate immune cells is terminated, *Foxp3* transcription may be initiated in antigen‐reactive T cells, as observed by *Foxp3*–Tocky 26, especially when adjacent T cells are proliferating and producing IL‐2, inducing the resolution of inflammation.

## Mechanisms for the consolidation and tuning of *Foxp*3 transcription – the role of autoregulatory transcriptional circuit for the *Foxp3* gene

The maintenance of *Foxp3* transcription in T_reg_ requires conserved non‐coding sequences 2 (CNS2), which includes the widely studied T_reg_‐specific demethylated region, TSDR 33. The cytosine–phosphate–guanine (CpG) motifs in the TSDR are methylated in non‐T_reg_ cells and fully demethylated in thymic T_regs_ 22, 33. The genetic deletion of CNS2 results in the reduction of Foxp3 expression in thymic T_regs_ but does not affect Foxp3 induction *in vitro* 32. CNS2 is bound by several key transcription factors, including the Runx/Cbf‐β complex 78, 79, 80, 81, Ets‐1 82, which makes an active complex with Runx1 83, Foxp3 protein 32 and STAT‐5 63.

Foxp3 binding to CNS2 is dependent upon Runx1/CBF‐β 32. Importantly, the expression of Foxp3 in T_regs_ is reduced in both CBF‐β‐deficient T_regs_ 78 and CNS2‐deleted T_regs_ 34. CNS2 is required for maintaining the number of T_regs_ in the periphery during homeostasis and is also important for sustaining Foxp3 expression during inflammation 7, 34. CNS2‐deleted T_regs_ lose Foxp3 expression in the presence of proinflammatory cytokines, including IL‐4 and IL‐6, and become effector T cells to enhance autoimmune inflammation in mice 7. Furthermore, analysis of TCR repertoires in human T_regs_ also suggests the dynamic regulation of both CD25 and Foxp3 on T cells in rheumatoid arthritis 84. These data together suggest that, although Foxp3 expression is commonly recognized to be stable, it is in fact dynamically regulated in Foxp3^+^ T_regs_ during homeostasis and during immune responses.

Our recent investigations using *Foxp3*–Tocky have shown that, intriguingly, resting T_regs_ have intermittent *Foxp3* transcription, while activated effector T_regs_ with high expression of immunoregulatory molecules (including CTLA‐4 and IL‐10) have more sustained *Foxp3* transcription throughout time 26. The phenotype of these effector T_regs_ with temporally persistent *Foxp3* transcription is in fact very similar to those of the effector T_regs_ that are dependent upon *Myb* 85 and the CD44^high^CD62L^low^ activated T_regs_ that are dependent upon TCR signals 35, which supports the model that TCR signals induce temporally persistent *Foxp3* transcription and thereby enhance the suppressive phenotype of T_regs_. Furthermore, by analysing female mice with heterozygosity for a hypomorphic Foxp3 mutant (namely, *Scurfy* mutation), Foxp3 protein sustains the temporally persistent *Foxp3* transcriptional dynamics that promote effector T_reg_ functions 26. In the thymus, the active demethylation of the TSDR occurs only after the initiation of *Foxp3* transcription and when *Foxp3* transcription is highly sustained over time 27. These indicate that Foxp3 protein and the *Foxp3* gene form an autoregulatory loop that consolidates the T_reg_‐type TSDR demethylation during thymic differentiation 27, and tunes *Foxp3* transcriptional activities and thereby activates their suppressive activity during inflammation 26. Given the critical roles of the Runx1/Cbf‐β complex in the maintenance of Foxp3 expression and the Foxp3–Runx1 interaction in T_reg_ differentiation and function, it is plausible that this autoregulatory transcriptional circuit is formed via the binding of Foxp3‐Runx1/Cbf‐β complex 32 to CNS2 of the *Foxp3* gene (Fig. 2).

## Dynamic regulation of epigenetic modifications and chromatin architecture of the *Foxp3 *gene

TCR‐induced *Foxp3* transcriptional activities can be opposed by epigenetic mechanisms for silencing *Foxp3* transcription. The SUMO E3 ligase Pias3 binds to the *Foxp3* promoter, and *Pias1* KO mice have increased frequencies of Foxp3^+^ cells in CD4^+^ T cells and reduced methylation of histone H3 at Lys9 (H3K9), which is a hallmark of repressed genes 86. The DNA methyltransferase DNA (cytosine‐5)‐methyltransferases (Dnmt1) and the high mobility group transcription factors Tcf1 and Lef1 constitutively repress *Foxp3* transcription in CD8+ T cells, as *Dnmt1* ^–/–^ or *Tcf1* ^–/–^ *Lef1* ^–/–^ double KO permits the differentiation of Foxp3^+^CD8^+^ T cells, which are rarely found in normal mice 87, 88. In addition, the induction of Foxp3 expression in *Dnmt1* ^–/–^ T does not require TGF‐β 87, suggesting that TGF‐β probably modulates epigenetic mechanisms in normal mice. Strong TCR signalling *in vitro* causes the accumulation of Dnmt1 at the *Foxp3* promoter, which can lead to increased CpG methylation and inhibition of *Foxp3* transcription 89. Thus, TGF‐β may be important for tuning Dnmt1 expression during T cell activation.

*Foxp3*–Tocky has shed light on the dynamics of *Foxp3* epigenetic regulation following the initiation of *Foxp3* transcription. Importantly, *Foxp3* transcription precedes the demethylation of TSDR in the thymus. Both thymic new *Foxp3* expressors, which are identified by Tocky 27, and immature CD24^high^FoxP3^+^CD4SP by *Foxp3*‐EGFP mice 90 have fully methylated TSDR. The active process for TSDR demethylation occurs only after *Foxp3* transcription is sustained over time and the Foxp3 autoregulatory loop is formed 26. Collectively, the interactions between Foxp3‐inducing and inhibiting factors occur during the early phase of T_reg_ differentiation when the *Foxp3* gene is still ‘silenced’, and we would therefore hypothesize that Foxp3 protein may also have roles in dynamically regulating the epigenetic modifications of the *Foxp3* gene. Future studies could therefore address the role of Foxp3 in the dynamic regulation of chromatin architecture, which can be investigated by chromatin conformation capture (3C) and derivative methods (e.g. Hi‐C). For example, the Zheng group showed that, using 3C, NFAT activation induces the interaction of the TSDR‐containing CNS2 with the *Foxp3* promoter, which facilitates enhanced *Foxp3* transcription 34. Using Hi‐C and clustered regularly interspaced short palindromic repeats (CRISPR)‐mediated mutation, the Zhao group showed that the mixed lineage leukaemia (MLL) family methyltransferase MLL4 binds to –8·5k upstream enhancer of the *Foxp3* gene, and makes a chromatin loop to promote the monomethylation of histone H3 at Lys4 (H3K4me1) in the promoter and CNS3, which activates *Foxp3* transcription 91. The chromatin organizing factor special AT‐rich sequence binding protein 1 (SATB1) is also involved in activating *Foxp3* transcription in the thymus, as the genetic deletion of SATB1 results in the marked reduction of Foxp3^+^ T_regs_ and the accumulation of thymic CD25^+^Foxp3^–^ T_reg_ precursors with reduced enhancer activity [which are identified by acetylation of histone H3 at Lys27 (H3K27ac)] 92. Thus, it is likely that chromatin remodelling of the *Foxp3* gene underlies the temporally dynamic *Foxp3* autoregulatory loop, suggesting that the former is also induced dynamically through the interactions between Foxp3 protein and key chromatin organizers and epigenetic regulators. In addition, as those chromatin organizers and epigenetic regulators control not only the *Foxp3* gene but also other genes, the chromatin remodelling of Foxp3‐target genes may be also induced dynamically in activated T_regs_ and differentiating T_regs_. Future studies, therefore, should investigate the role of Foxp3 protein and its co‐factors in the temporally dynamic regulation of chromatin structure within and outside the *Foxp3 *gene region.

## Dynamic *Foxp3* expression *in vivo*: perspectives for basic immunology and clinical relevance

After the emergence of single‐cell technologies and the Tocky tool, studies on T cell regulation are shifting from the stability and plasticity of T_reg_ to the investigation of temporal changes in Foxp3‐mediated mechanisms *in vivo*. Our analysis of T_regs_ in peripheral immune compartments show that, in non‐inflammatory conditions, *Foxp3* transcription is most probably modelled by intermittent gene activity 26. This intermittent transcription may offer an explanation for the low frequency of T_reg_ cells with detectable *Foxp3* transcripts in T_reg_ cells analysed by single‐cell RNA‐seq 93, 94, although these data sets have limitations due to shallow sequencing depths. Given that the temporal changes in *Foxp3* transcription control T_reg_ function and effector T_reg_ differentiation, future work will investigate the molecular mechanisms that control the real‐time transcribing of the *Foxp3* gene, which can be analysed by the Tocky system. In addition, in line with the temporally dynamic regulation of *Foxp3* transcription *in vivo*, the significance of thymic and peripheral T_reg_ markers needs to be readdressed. Our investigation using *Foxp3*–Tocky has confirmed that the expression of Neuropilin 1 95 and Helios 96 are dynamically regulated in T_regs_ according to *Foxp3* transcription dynamics 26, and therefore are not faithful markers of thymic T_regs_, as has been noted previously in the literature 97.

Importantly, clinical studies and immunotherapy development may be benefited by the endorsement of the dynamic perspective. Whether targeting T_regs_ or not, immunotherapy may dynamically change *Foxp3* transcription. If these dynamic responses are clarified, immunotherapy targeting T cells may be better designed with a more tailored strategy, as we recently showed by manipulating *Foxp3* transcriptional dynamics through targeting inflammation‐reactive effector T_regs_ by OX40 (CD134) and tumour necrosis factor receptor II (Tnfrsf1b which are expressed specifically in T_regs_ with temporally persistent *Foxp3* transcription 26. We therefore envisage that the investigation of dynamic changes in molecular mechanisms during T cell responses *in vivo* will improve the predictability of preclinical studies and thereby contribute to the development of new immunotherapies for autoimmune and cancer patients.

## Disclosures

The authors declare that no conflicts of interest exist in relation to this paper.
